# Stress behaviours buffer macaques from aggression

**DOI:** 10.1038/s41598-017-10754-8

**Published:** 2017-09-11

**Authors:** Jamie Whitehouse, Jérôme Micheletta, Bridget M. Waller

**Affiliations:** Department of Psychology, Centre for Comparative and Evolutionary Psychology, University of Portsmouth, Portsmouth, UK

## Abstract

Primates (including humans) scratch when stressed. So far, such scratching has been seen as a by-product of physiological processes associated with stress, and attributed proximate, regulatory function. However, it is possible that others could use this relationship between scratching and stress as an indication of the animal’s stress state, and thus scratching could potentially have social function. As a test of this theory, we measured the production of, and social responses to scratching in a group of free-ranging rhesus macaques (*Macaca mulatta*). Firstly, we found that the likelihood of scratching was greater around periods of heightened social stress, such as being in proximity to high-ranking individuals, or non-friends. Secondly, when macaques scratched, subsequent interactions were less likely to be aggressive and more likely to be affiliative. Potential attackers may avoid attacking stressed individuals as stressed individuals could behave unpredictably or be weakened by their state of stress (rendering aggression risky and/or unnecessary). Observable stress behaviour could therefore have additional adaptive value by reducing the potential for escalated aggression, benefiting both senders and receivers by facilitating social cohesion. This basic ability to recognise stress in others could also be an important component in the evolution of social cognition such as empathy.

## Introduction

Stress is manifest in the behaviour of animals^1, 2^. Stress is a biological response to the physical and physiological challenges animals face in their environment, and often specifically refers to a disruption of an animal’s homeostasis^3, 4^. A stress response can be elicited by many types of physical stressors, and also social factors such as competition for resources or conflict with others^2, 5^. There are strong links between the physiological markers of stress (such as raised cortisol level) and behaviour^6^. One key behavioural correlate of stress, common particularly within the primates, is scratching (i.e the repetitive raking of the skin on face and/or body, with the fingers of the hand or feet)^7^. For example, scratching is often increased in victims of intense conflict^8^, or in mothers who are separated from their newborn offspring^9^. In addition, as the difficultly of cognitive tasks presented to chimpanzees increases, so does the rates of self-directed behaviours including scratching^10, 11^. Rates of scratching can be both increased and decreased experimentally in macaques through the administration of anxiogenic and anxiolytic drugs respectively^2, 6^. On the whole, therefore, the evidence suggests a link between the experience of stress, the physiology of stress, and scratching.

Scratching is usually interpreted as a by-product of physiological responses^12^, sometimes with proximate value attributed to internal regulatory processes^13–15^. For example, it has been argued that scratching may distract individuals from the stressful stimulus and/or reduce the negative arousal associated with stressful events^16^. However, given the overt visual nature of scratching, there is also potential for these behaviours to alert others to the state of the scratcher, and therefore have communicative function within a social environment. Scratching could act as a cue^17^ if other individuals take advantage of the association between the scratching behaviour, and other aspects of the scratching individual that could indicate stress. If selection has acted on the scratching behaviour to shape its form and function, scratching could also gain signal function over evolutionary time and be considered an independent signal^18^. Indeed, a number of researchers have proposed that these behaviours could have communicative value^2, 11, 19^. For example, displacement activities have been described in many animals, where a seemingly irrelevant behaviour is incorporated into a display which increases the salience to others (e.g preening as part of the sexual displays of some bird species^1^). Through learned or evolved associations between stress and behaviour^20^, an audience could gain valuable and reliable information about the scratching individual. This could in turn motivate observers to direct (or avoid) interactions with the scratching individuals. Such a sensitivity to the internal or motivational states of others, including the experience of stress, would be favoured by natural selection as this would allow individuals to better navigate a social environment by informing their interactions with others^18^. An alternate suggestion is that stress behaviours could be produced to detract attention away from more salient cues about internal state in order to conceal information that could expose weakness^2, 19^. Evidence to support these claims, however, is lacking and/or anecdotal. In addition, it could be argued that producing behaviours so closely linked to stress could be a maladaptive strategy if it exposes cognitive and physical weakness. An empirical demonstration of how stress behaviours are perceived and responded to by others would therefore be helpful in understanding why behaviours linked to stress have been selected for during evolution.

We know that, at least to some degree, some social animals can be responsive to the emotional experiences of others^21^ and have even been attributed empathy-like responses^22, 23^. We also know that affiliative post-conflict interactions towards victims are common^24^, from both the aggressor^25, 26^ and from bystanders^27^. However, the specific behaviours that elicit these kinds of responses are difficult to identify. Indeed, determining whether the responses are made in reaction to the stressful event itself or to the behaviours or events associated with stress is difficult to determine. For phenomena like *consolation* ^28^ or *empathy* to develop, however a basic ability to recognise stress and other negative emotional experiences in others through their behaviour could be useful. It could be that these behaviours are used for their predictive value about the scratcher’s potential actions^29, 30^. Stress can influence the subsequent decision making and behavioural responses of animals^31^, for example primates can be more unpredictably aggressive towards others when stressed^32^. Considering how stress behaviours can be used by other individuals as potential cues to future behaviour rather than just by-products of an internal state could therefore provide us with a more appropriate framework to begin exploring their adaptive value^33^. Such approaches have been fruitful in the study of other communicative behaviours, such as facial expressions^29, 30^.

Here, we examine the social function of stress behaviours in a group of free-ranging rhesus macaques (*Macaca mulatta*), focusing on a stress behaviour commonly documented in macaques and many other social primates; scratching^2, 6^. Macaques live in complex societies underpinned by communicative systems that facilitate both cooperative^34^ and competitive^35, 36^ efforts between individuals. The extensive communicative repertoire (which include a huge amount of facial expressions, gestures, and vocalisations^35^) in these species therefore makes the macaques a good model for the study of communication. We used a multi-model inference approach, first to explore which social factors, if any, best predict the production of scratching behaviours in the macaques. To do this we looked at the social relationships between the scratcher and their neighbouring individuals, to confirm whether potentially stressful situations are more likely to elicit scratching. Secondly, we explored how the presence of scratching behaviours, among other social factors, modulates future social interaction. If others can use scratching behaviours as cues to potential future behaviour, we expected to see a difference in the type of response we observed after their occurrence. If for example, scratching is less likely to be followed by conflict, this could demonstrate a key adaptive advantage to both producing stress-associated behaviours and responding to them.

## Results

**Figure 1:**
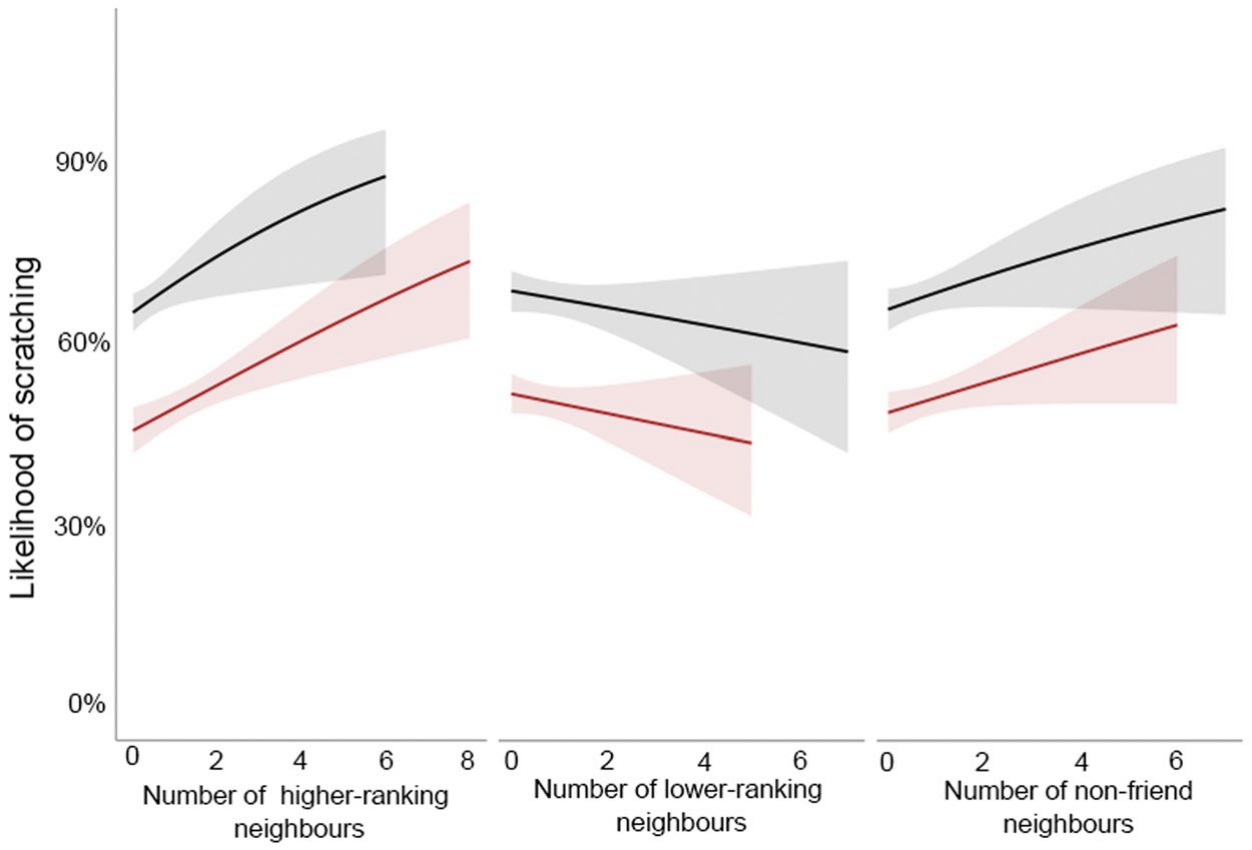
Production of scratching. The predicted probability of scratching in the presence of higher-ranking individuals (left), lower-ranking individuals (middle) and non-friends (right). Males are in black, and females are in red. Probabilities extracted from models including only the sex of the focal, the factor in question, and random effects. Shaded areas represent 95% confidence intervals.

We compared several models that included a range of social variables to assess which social factors best explained the production of scratching behaviours (Tables 1 and 2, and a full breakdown of these models can be found in the *Methods*). We considered all models within a **∆**AICc of <2 as strongly competing, and those within <4 as weakly competing. Any model with a **∆**AICc above this, we considered to be a weaker model fit^37^. All parameter estimates and standard errors have been averaged across all models (full-averaging^37^). The top ranked model, which included the presence of higher-ranking individuals as a single factor, had the lowest AICc value, and highest Akaike weight of 0.374. In this model, individuals were more likely to scratch when surrounded by higher-ranking individuals (Fig. 1, β = 0.124, SE = 0.069). A closely competing model included the presence of higher-ranking individuals and non-friends (i.e a high social stress model, **∆**AICc = 0.97, *w* = 0.231, ER = 1.62). Individuals were also more likely to scratch when surrounded by non-friends (Fig. 1, β = 0.025, SE = 0.05). Each of these models supports the production of scratching as a marker of social stress^2^. To be cautious, we can also consider a full dominance model containing both the presence of high-ranking individuals, and the presence of low-ranking individuals (**∆**AICc = 2.01, *w* = 0.137, ER = 2.73). In contrast to the effect of high-ranking individuals on scratching, the presence of lower-ranking individuals reduced the occurrence of scratching behaviours (Fig. 1, β = −0.0002, SE = 0.019). A full social model containing each of the social factors described above, including the presence of friends was weakly competing against these models (**∆**AICc = 2.43, *w* = 0.111, ER = 3.37), as well as a model containing only the presence of non-friends (**∆**AICc = 3.68, *w* = 0.059, ER = 6.29). The above models made up 0.912 of the accumulative weight during model selection. Models that included the number of neighbours as a factor did not fit well to the data (**∆**AICc > 4); instead it appears quality and type of social relationship with neighbours has a greater influence than quantity of neighbours on the production of scratching. When we directly compare our high social stress and low social stress models, we find that high social stress factors (presence of higher-ranking individuals, non-friends) to be more predictive of scratching behaviour then low social stress factors (presence of lower-ranking individuals, friends, and relatives **∆**AICc = 8.96). Overall, males scratched more than females (*β* = 0.891, SE = 0.172).

**Table 1:** Model characteristics. Models are ranked by the AICc value (lowest to highest; best to worse fit).

Models	df	logLik	AICc	**∆**AICc	*w*	Acc. *w*	ER
***Production of scratching***							
Number of higher-ranking	5	−1660.06	3330.14	—	0.374	0.374	—
High social stress	6	−1659.54	3331.11	0.97	0.231	0.605	1.62
Dominance	6	−1660.06	3332.15	2.01	0.137	0.742	2.73
Social	7	−1659.26	3332.57	2.43	0.111	0.853	3.37
Number of non-friends	5	−1661.90	3333.82	3.68	0.059	0.912	6.29
Number of neighbours	5	−1662.46	3334.94	4.80	0.034	0.946	11.01
Friendship	6	−1661.65	3335.32	5.19	0.028	0.974	13.37
Kinship	5	−1663.09	3336.20	6.06	0.018	0.993	20.68
Number of friends	5	−1665.00	3340.02	9.89	0.003	0.995	>38
Low social stress	7	−1663.01	3340.07	9.93	0.003	0.998	>38
Number of lower-ranking	5	−1665.22	3340.47	10.33	0.002	1.000	>38
***Likelihood of aggression***							
Scratch-social	7	−201.45	417.19	—	0.555	0.555	—
Full	8	−200.70	417.77	0.59	0.414	0.969	1.34
Social	6	−205.37	422.96	5.77	0.031	>0.999	17.91
Scratch-dominance	6	−212.00	436.22	19.03	4.09*e* ^*−5*^	>0.999	>55
Dominance	5	−216.31	442.78	25.60	1.53*e* ^*−6*^	>0.999	>55
Scratch-friendship	6	−220.10	452.42	35.23	1.24*e* ^*−8*^	>0.999	>55
Friendship	5	−224.59	459.33	42.14	3.92*e* ^−10^	>0.999	>55
Scratch-kin	6	−229.57	471.36	54.18	9.54*e* ^−13^	>0.999	>55
Scratch	5	−233.89	477.93	60.74	3.58*e* ^*−14*^	>0.999	>55
Kinship	5	−235.09	480.34	63.16	1.07*e* ^−14^	>0.999	>55

Df = Degrees of freedom, LogLik = Log-likelihood, AICc = Akaikes Information Criterion corrected for small sample sizes, **∆**AICc = the difference in AIC between the highest ranked, and target model, *w* = Akaikes weight, Acc. *w* = the cumulative weight between the target model and the highest ranked model, ER = Evidence ratio (the weight of the high-ranked ranked model divided by the target model).

**Table 2:** Model averaged parameters. The parameter estimate, and standard error for each factor in both analyses. Estimates are averaged through *full averaging*.

Factors	Estimate	SE
***Production of scratching***		
(Intercept)	−0.195	0.130
Sex	0.891	0.172
Number of Higher ranking	0.124	0.069
Number of non-friends	0.025	0.050
Number of lower ranking	−0.0002	0.019
Number of friends	−0.005	0.026
Number of neighbours	0.003	0.016
Number of kin	0.008	0.061
***Likelihood of aggression***		
(Intercept)	−0.132	0.390
Sex	0.851	0.401
Scratch	−1.256	0.534
Friendship (logCSI)	−1.600	0.403
Rank difference	−0.025	0.005
Kinship	−0.844	1.511

**Figure 2:**
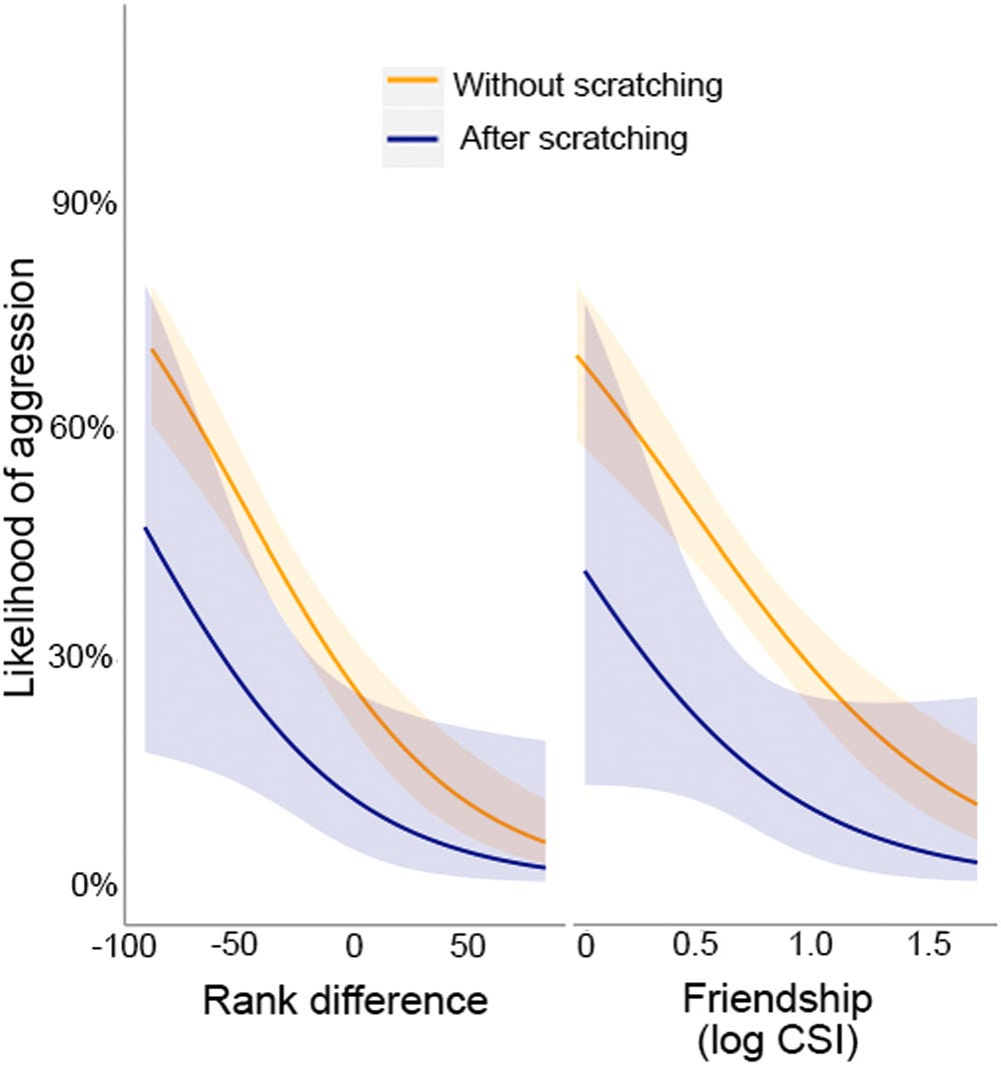
Likelihood of received aggression post-scratch. This graph shows the probability of a received interaction being aggressive, depending on if it occurred post-scratch or not. Probabilities extracted from models including only the sex of the focal, the factor in question, and random effects. This is compared against the rank difference on the left, and the degree of friendship (log CSI) on the right. A positive rank difference implies the focal is ranked higher than the actor a negative rank difference implies the focal is ranked lower than the actor. A higher CSI represents a stronger social bond. Shaded areas represent 95% confidence intervals.

To assess how scratching was responded to by others, we looked at all social interactions received by the scratching individual, separating those which were affiliative (0) and those which were aggressive (1). We then compared several models that included scratching and a range of social variables to assess which factors best explained the likelihood of future aggression (Tables 1 and 2, a full breakdown of these models can be found in the *Methods*). The highest ranked model, the Scratch-social model, which included measures of relationship quality (Friendship; as measured through a composite sociality index^38^, and rank difference; calculated from ELO ratings^39^) as well as the presence of scratching prior to the interaction, had the lowest AICc value, and highest Akaike weight of 0.555. This was followed by a full model, which additionally included measures of kinship (**∆**AICc = 0.59, *w* = 0.141, ER = 1.34); however this model fit was likely inflated by the fact the scratch-social model is nested within the full model, as otherwise, models that included kinship had very poor fit in comparison (**∆**AICc > 54). Within the Scratch-social model, friendship and rank difference both affected the likelihood of an interaction being aggressive or not (Fig. 2). The likelihood of aggression increased as friendship decreased (*β* = −1.596, SE = 0.403), and the likelihood of aggression increased as rank difference decreased (ie. individuals were more likely to aggress those ranked lowered than them, *β* = −0.025, SE = 0.005). Interestingly, this was further modulated by the presence of a scratch; when a scratch occurred prior to the interaction, the likelihood of aggression was lower (Fig. 2, β = −1.256, SE = 0.534). The two above models made up 0.969 of the accumulative weight during model selection. When we further compare models within the candidate set, we find the inclusion of scratching with other social factors improved the fit of models in all cases - Scratch-social vs Social model, **∆**AICc = 5.77; Scratch-dominance vs Dominance model, **∆**AICc = 6.56; Scratch-friendship vs Friendship model, **∆**AICc = 6.91; Scratch-kinship vs Kinship model, **∆**AICc = 8.98.

## Discussion

When macaques were in the presence of non-friends or higher-ranking individuals, they scratched more, providing further evidence for the link between social stress and scratching behaviours. Macaques were also less likely to be the target of aggression after they scratched. Scratching, therefore, appeared to elicit an immediate social response from others and seemed to have a broad function of modulating aggression and promoting non-aggressive interaction. This is the first data to suggest that scratching can be detected and responded to socially by others, and provides some evidence towards a communicative function.

We know that macaques appear less stressed (and produce less stress-behaviours) after a clear dominance relationship has been established between individuals^40^. Presumable, after a hierarchy has been established the actions of others become more predictable^2^. Here, stress behaviours are occurring more frequently around individuals with whom they are less bonded, probably because the likelihood of conflict is higher and/or because the outcomes of any interactions are less predictable, and thus more stressful. Candidate models which included the presence of important social partners such as friends^38^ and kin were less explanatory of scratching. On a whole, therefore, these data provides little evidence that the production of scratching is directed as a communicative signal, but instead corroborates with other data that scratching is a marker of social stress^2^.

When scratching occurred before social interactions, the likelihood of a subsequent aggressive interaction was reduced. Conflict is extremely costly, in both energy expenditure and risk of injury^41^, and behavioural strategies to avoid and reduce physical conflict are common throughout gregarious animals^42, 43^. Recent experimental research has suggested that presence of *any* emotional facial expressions in primates predicted a reduced likelihood of subsequent aggressive interactions^44^. Therefore, it might not be the specific emotional content of facial expressions that is important to others, but more that they indicate *something* about future behaviours and thus reduce uncertainty in observers. Scratching could be conceptualised similarly, as a reliable cue indicative of potential future behaviour, such as an individual’s potential for unpredictable or aggressive behaviour^32^. Recognising stress in others through behaviour such as scratching could function to help anticipate the potential future behaviour of others, allowing for more coordinated interaction and reducing the need for conflict. This could be of great adaptive value in animals with high degrees of sociality such as primates, of which many are dependent on a cohesive social environment.

It is important, however, to be cautious in the interpretation of these data. Although we have attempted to reduce confounding variables by reducing our dataset to social interactions that are isolated from other behaviours, it is extremely difficult to know with absolute certainty that it is the scratch which is being responded to, and that we haven’t missed more subtle phenomena affecting the behavioural responses in others. In addition, although the current published evidence connecting scratching to stress is convincing^2^, self-directed behaviours are extremely complex and have been documented in other behavioural contexts such as behavioural transitions^45^ and positive emotional arousal^46^. Scratching has also been interpreted as a potential gesture during affiliative interactions in chimpanzees, implying that their production can be intentional^47^. Further experimental cognitive research, which aims to probe how primates perceive scratching and relate it to context would help better understand primates’ perception of these behaviours. For example, we can test whether macaques view scratching as aversive and has to be avoided, or whether they truly understand the association with internal stress.

These findings suggest that stress-related behaviours are potentially functional, not only for the regulation of internal states, but also in communication with others. This could help stimulate new approaches to stress by situating stress within a social interaction rather than focussing on the individual alone. Such an approach would not only impact our fundamental understanding of stress and the evolution of stress in humans, but also the strategies we employ to manage stress in captive animals. We emphasise the necessity to broaden our study of emotional behaviour to include a more adaptationist framework. We also support the view that behaviours and expressions are not only rooted in the internal state of the senders but can also be conceptualised as indicators of how individuals are likely to behave in the future^29, 30^. By revealing stress, and thus future behaviours and intentions more transparently, animals can ultimately reduce the necessity for conflict and therefore promote a more cohesive social group. Crucially, this basic ability to recognise stress in others could be an important component of social cognition such as emotional perception, and empathy.

## Methods

### Subjects and study site

We studied a group of free-ranging rhesus macaques (*Macaca mulatta*) in Cayo Santiago (Punta Santiago, Puerto Rico) between June and November 2016. Our subject group (V) consisted of 114–118 individually recognised adult monkeys and approximately 110 unidentifiable juveniles at the time of the study. Data were collected during the birthing season, and therefore the number of infants in the group varied. Animals were provisioned daily with commercial monkey pellets, and natural vegetation and water was available *ad libitum*.

### Data Collection

Monkeys were followed between 7.00 am and 2.30 pm, 6 days per week. Data were collected on 45 adult macaques (21 males, 24 females) using focal animal and instantaneous scan sampling^48^. Identities of animals could be confirmed by tattoos on the chest and thigh, as well as systematic ear notches. Focal follows were performed over 30 minute periods in a randomised order and instantaneous scans were performed every 5 minutes within the focal. If an individual went out of view for 10 minutes of the follow, the follow was discarded and the next individual was followed. All behavioural data were recorded on a Samsung Galaxy Tab 4 installed with Prim8 software^49^, a free, live behavioural data collection tool for Android based systems which allows a user to record continuous and scan data simultaneously (http://www.prim8software.com/)^49^. We recorded all instances of scratching and all affiliative and aggressive social behaviours^35^, see Table 3. For all social behaviours the actor and the receiver were identified. The identities of all nearby individuals to the focal animal (within 0–3 m) were recorded in the instantaneous scan samples. Whenever possible, follows on all focal individuals were conducted before any individual was repeated. We conducted 10 follows on each focal individual. Taking into account the time the animals spent out of view of the researcher, this resulted in an average of 280.6 (±16.8 SD) minutes of observation time per animal.

**Table 3:** An ethogram of the stress and social behaviours which were recorded, along with operational definitions of each behaviour.

Behaviour	Operational definition
***Stress-behaviours***	
Scratching	The repetitive raking of the skin, with the fingers of the hand or feet^7^
***Affiliative Behaviours***	
Social-grooming	Grooming/cleaning of the hair on other individual with the hands or mouth^7^. Used in hygienic contexts, and during the maintenance of social bonds.
Lip-smacking	Lips are pursed, and lower jaw moved rapidly. Often made up of other visual and auditory components (eg. flattening of ears, head-turns, soft grunting^34^)
Silent-bared-teeth	Both lips retracted to reveal the teeth, often accompanied by a raised scalp and flattened ears. An affiliative signal, but sometimes used as a submissive response to threats^7^.
Approaches	An individual moves towards a social partner.
Embrace/contact-sitting	An individual sits in contact with the partner, may include grasping of the hair^7^.
***Aggressive Behaviours***	
Non-contact aggression	Includes aggressive chasing or lunging.
Contact aggression	Includes biting, grabbing, and slapping. Usually following a chase.
Open-mouthed threat	The mouth is half-opened, accompanied by a raised brow and staring. Often includes a rattle vocalisation^7^.
Displacement	An individual moves towards another individual, whom then subsequently walks away. A reliable cue of dominance^7^.

### Measures of relationship quality

To explore the social function of stress behaviours, we needed to take into account the quality of the social relationship during interactions. For each dyad we calculated the strength of the social bond, a difference in competitive success, and a coefficient of relatedness.

To estimate the strength of social bonds between pairs of individuals in the group we calculated a composite sociality index (CSI^38^), often used as a measure of friendship in animals. This measure is based on two affiliative factors; the frequency of scans where individuals were found in a close proximity, and the frequency of scans where individuals were found engaged in grooming. This is calculated through the following equation:

$$\frac{(\frac{Gab}{\mu G}+\frac{Pab}{\mu P})}{2}$$

where *Gab*, is the frequency with which dyad ab can be observed grooming, and μG is the mean frequency of grooming for all dyads, and *Pab* is frequency in which dyad ab can be observed within a close proximity (<3 meters), and μP is the mean frequency of proximity for all dyads. This CSI index allows us to characterise the strength of a social bond relative to the rest of the group. As CSI data tends to be positively skewed, this data was log-transformed to an approximate normal distribution before being used in analyses.

To estimate the difference in competitive success, we calculated an ELO-rating for each individual (R package: EloRating^39^). In this analysis, all individuals begin with an equal rating, which is then adjusted based on the outcome of an interaction. We looked at outcomes of all observed conflict, and all observed displacement interactions. Winners ratings increase as losers ratings decrease, with the magnitude of change reflecting the expected outcome (e.g. a lower rated individual winning against a high rated individual will result in a higher magnitude of change). The final ELO-rating of adults was converted to an absolute rank (from 1–99, this range is smaller than the total number of adults in the group as some non-focals were never seen engaged in competitive behaviours). The absolute rank of one individual was subtracted from another to provide a rank difference.

Maternal relatedness was known, but paternal relatedness was not. Maternal relatedness was quantified through a *coefficient of relatedness (r)* index that represents the probability that two individuals will have copies of the same gene^50^. Mother-offspring pairs have an *r* of 0.5, grandmother-grandchildren pairs have an *r* of 0.25, siblings have an *r* of 0.25, and maternally unrelated individuals have an *r* of 0.

### Statistical analysis

Two candidate sets of models were produced for analysis, one to assess the factors that affected the production of scratching (Table 4), and one to assess which factors (including scratching) affected the likelihood of future aggression (Table 5). Data were applied to generalized linear mixed-models with a binomial error structure and logit link function, applying random intercept/slope models. Models were produced using *glmr* function, in the *lmer4* package for R studio (R version 3.31^51^). For both candidate sets of models, we use multi-model inference approach using Akaike’s information criterion to assess the influence of each of the factors. This approach allows for the comparison of multiple potentially competing models simultaneously, providing an approximation that any given model in a set is the best whilst accounting for uncertainty in model selection. Further inferences can be then based on a range of competing models^37^. Such a multi-model inference approach is growing in popularity throughout the study of behaviour and ecology. In this approach, models are judged and ranked based on their AICc value, (Akaike’s information criterion corrected for small sample size), where a smaller AICc value signifies a better model fit, and a smaller difference between the AICc of different models signifies when two models are competing^37^. From AICc values, Akaike weights (*w*) for each model is calculated, a value which represents the probability that the model under consideration is the best approximating model within the set, with strongly fitting models tending towards a *w* of 1, and weakly fitting models tending towards a *w* of 0. Model selection, calculation of AICc values, and Akaike weights were all calculated using the function *mod.sel*, in the package *MuMIn* for R. Finally, parameter estimates are averaged across all models (full averaging^37^), to assess the relative contributions of the factors within competing models. Model averaging was calculated using the function *mod.avg* in the package *MuMin*. All full models were tested for multicollinearity (function *vif*, package *car*) and over-dispersion (function *dispersion_glmer*, package *blmeco*). Additionally, residuals were visually inspected for extreme deviation from normality. All models had a low multicollinearity (variance inflation factor <2) and showed no evidence for over-dispersion.

**Table 4:** Candidate model set 1.

**Candidate model**	Factors					
	**nb of Neighbours**	**nb Friends**	**nb of Non- friends**	**nb of Higher ranking**	**nb of Lower ranking**	**nb of relatives**
Number of neighbours	1	0	0	0	0	0
Social	0	1	1	1	1	0
High social stress	0	0	1	1	0	0
Low social stress	0	1	0	0	1	1
Dominance	0	0	0	1	1	0
Friendship	0	1	1	0	0	0
Number of friends	0	1	0	0	0	0
Number of non-friends	0	0	1	0	0	0
Number of higher-ranking	0	0	0	1	0	0
Number of lower-ranking	0	0	0	0	1	0
Number of relatives	0	0	0	0	0	1

Numbers represent inclusion of factor in the model (1) or not (0). Response variable: Occurrence of scratching. Sex of the focal was included in all models. Focal ID and follow ID were included as random factors in all models.

**Table 5:** Candidate model set 2.

**Candidate model**	*Factors*			
	**Scratch**	**CSI**	**∆ELO**	**Kin**
Full	1	1	1	1
Scratch	1	0	0	0
Social	0	1	1	0
Scratch-social	1	1	1	0
Friendship	0	1	0	0
Scratch-friendship	1	1	0	0
Dominance	0	0	1	0
Scratch-dominance	1	0	1	0
Kinship	0	0	0	1
Scratch-kinship	1	0	0	1

Numbers represent inclusion of factor in the model (1) or not (0). Response variable: Likelihood of receiving aggression. Sex of the focal was included in all models. Focal ID and actor ID were included as random factors in all models.

For our first analysis, we separated each 30-minute follow into six 5-minute observation periods. For each observation period we measured if scratching was present (did occur (1), or did not occur (0)), how many neighbouring individuals were in proximity to the focal during this period (within 0–3 metres), the number of neighbours that were friends, the number of neighbours which were non-friends, the number of neighbours that were higher-ranking individuals, the number of neighbours that were lower ranking individuals and the number of neighbours which were maternal relatives. We defined friends as dyads with a CSI higher than the 3^rd^ quartile + 1.5 x the interquartile range (i.e the outlier rule^52^), and non-friends as individuals below this. As the number of neighbours was directly associated with other factors (e.g number of friends, number of higher-ranked individuals), these factors were not included together in the same models to maintain low multicollinearity. To control for differences between sexes, the sex of the focal was included as a control in all models. The identification of the focal, and the focal follow that the observation period was extracted from were included as random factors in all models to avoid pseudoreplication^53^. The candidate models that were built from these factors and applied to the model selection process are found in full in Table 4.

For our second analyses, we looked at all received social interactions in our dataset, separating those which were aggressive from those which were affiliative (see Table 3). The likelihood of receiving aggression was set as our binomial response variable (receiving aggression (1) or not (0)). Only social interactions that were isolated from other interactions (ie. no other social behaviour occurred between the actor and receiver within 1 minute prior) were included in the analyses. This was to control for the potential effect of other variables outside of our chosen factors (e.g. other communicative behaviours). We measured whether a scratch occurred within the 30 seconds prior to an interaction (did occur (1), or did not (0)) and included this as a factor in our model selection process. We additionally looked at the effects of friendship (the strength of a social bond; CSI), rank difference (calculated from ELO ratings), and the maternal relatedness of individuals (related (1), or not (0)) on the likelihood of aggression. To control for differences between sexes, the sex of the focal was included as a control in all models. The identification of the focal animal, and the identification of the actor were included as random factors in every model. The candidate models that were built from these factors and applied to the model selection process are found in full in Table 5.

## Ethical Note

This study received approval from the Animal Welfare and Ethical Review Body (AWERB), University of Portsmouth. All methods were in compliance with the ASAB/ABS guidelines for the use of animals in research.

### Data Availability

The datasets generated and analysed during the current study are available from the authors upon reasonable request.
